# Supplementary material for: Perceived Impact of Taiwan’s National Health Insurance Allocation Strategy: Health Professionals’ Perspective
Source: Int J Environ Res Public Health. 2019 Feb 5;16(3):467. doi: 10.3390/ijerph16030467 (PMC6388230; doi:10.3390/ijerph16030467)
Supplement: Supplementary file 1 [file ijerph-16-00467-s001.pdf]

Supplementary Materials

Table 1. Covariance Matrix of Latent Variables .

	PPIAM	PNIAM	OPS	ARAM	BNHCRAM
**PPIAM**	1.73				
**PNIAM**	-0.01	1.66			
**OPS**	0.87	-0.37	1.04		
**ARAM**	-0.04	-0.01	-0.02	0.06	
**BNHCRAM**	0.27	-0.01	0.27	-0.03	0.28

Table 2. Model fit statistic and the criteria used for evaluating the structural model.

Model fit	Model fit indices
Absolute Fit Indices:	
χ^2 (*df*)	2495 (290)
***p*-value**	< .001
**Goodness-of-fit index (GFI)**	0.61
**Adjusted GFI (AGFI)**	0.53
**Root-mean-square error of approximation (RMSEA)**	0.08
**Root-mean-square residual (RMR)**	0.16
**Standardized RMR (SRMR)**	0.11
Incremental Fit Indices:	
**Normed fit index (NFI)**	0.89
**Non-normed fit index (NNFI)/Tucker-Lewis**	0.92
**Comparative fit index (CFI)**	0.92
**Incremental fit index (IFI)**	0.92
**Parsimony GFI**	0.50
